# CatchU. . . Before You Fall: A Quantitative Digital Health App for Predicting and Monitoring Falls in Older Adults

**DOI:** 10.1093/geroni/igaf122.1767

**Published:** 2025-12-31

**Authors:** Jeannette Mahoney, Claudene George, Joe Verghese

**Affiliations:** Stony Brook University, Stony Brook, New York, United States; Albert Einstein College of Medicine, Bronx, New York, United States; Stony Brook University, Stony Brook, New York, United States

## Abstract

Thirty-three percent of Americans aged 65 and over will fall each year at an annual cost of over $50 billion to US healthcare. Falls are the leading cause of injury & injury-related death among older adults. In fact, over 3 million older adults will require an ED visit because of fall-related injuries. People living with Alzheimer’s disease (AD) are at even greater risk for mobility declines and falls. Our research suggests that the magnitude of multisensory integration, which is crucial for independent functioning & completion of daily activities, is linked to falls, balance, gait, cognition and amyloid pathology. The CDC recommends routine fall-risk screening annually, but this is not well-integrated into clinical practice. There is a large unmet need for quantitative, research-based digital health tools that raise falls awareness, while monitoring and improving wellness - especially for high-risk older adults living with preclinical Alzheimer’s disease. CatchU®…Before You Fall, a 10-minute multisensory digital health assessment grounded in over 15 years of research, uniquely fills this void by assessing visual-somatosensory integration performance in conjunction with existing medical comorbidities, in an effort to detect the propensity of falling in the near future. The vision and mission of CatchU® is to alleviate disability, promote independence, and increase quality of life for older adults and all people by promoting provider-initiated falls counseling and easy access to personalized recommendations scientifically proven to reduce falls.

